# Supplementary material for: Impact of co-morbid common mental disorder symptoms in people with epilepsy in Ethiopia on quality of life and functional disability: a cohort study
Source: Glob Ment Health (Camb). 2025 Feb 26;12:e33. doi: 10.1017/gmh.2025.24 (PMC11949734; doi:10.1017/gmh.2025.24)
Supplement: Tsigebrhan et al. supplementary material 1 — Tsigebrhan et al. supplementary material [file S205442512500024Xsup001.pdf]

Supplementary file 1

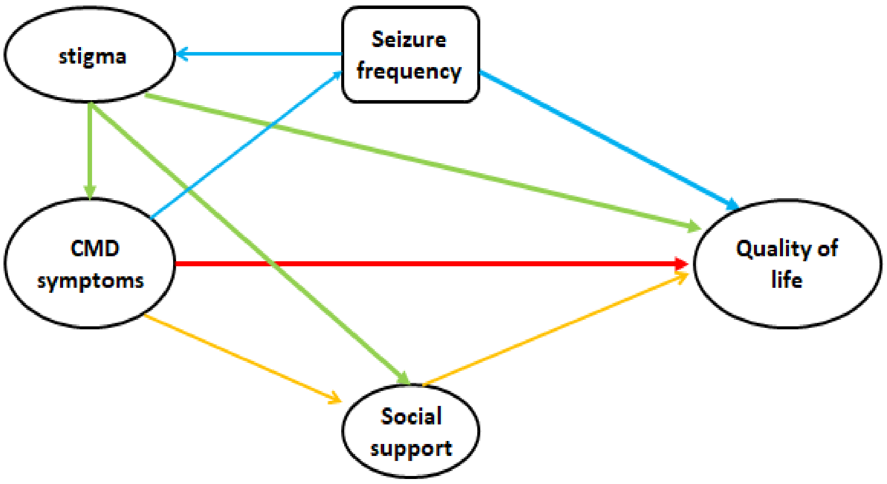

Figure 2. Hypothesised direct and indirect pathways linking co-morbid common mental disorder symptoms with quality of life
