# Supplementary material for: Impact of co-morbid common mental disorder symptoms in people with epilepsy in Ethiopia on quality of life and functional disability: a cohort study
Source: Glob Ment Health (Camb). 2025 Feb 26;12:e33. doi: 10.1017/gmh.2025.24 (PMC11949734; doi:10.1017/gmh.2025.24)
Supplement: Tsigebrhan et al. supplementary material 2 — Tsigebrhan et al. supplementary material [file S205442512500024Xsup002.pdf]

Supplementary table 1 Baseline characteristics between those lost to follow up and remained in the cohort

Characteristics		Remained N=219 (n, %)	Lost to follow up N=18 (n, %)	Fisher exact test or Wilcoxon rank sum test, p value
Gender	Male<br>Female	132 (60.3)<br>87 (39.7)	8 (44.4)<br>10 (55.6)	0.19
Age	In years	Median 32<br>(IQR 22, 42)	Median 26.5<br>(IQR 20, 38)	0.83
Residence	Urban<br>Rural	26 (11.9)<br>193 (88.1)	3 (16.7)<br>193 (83.1)	0.47
Marital status	Married<br>Single, divorced or widowed	118 (53.9)<br>101 (46.2)	5 (27.8)<br>13 (72.2)	**0.05**
Relative wealth	Average or above<br>Low or very low	64 (29.2)<br>155 (70.8)	4 (22.2)<br>14 (77.8)	0.79
Stressful life events	Number in the past 6 months	Median 0<br>(IQR 0, 1)	Median 1<br>(IQR 0, 3)	**0.04**
Comorbid mental disorders	No<br>Yes	193 (88.1)<br>26 (11.9)	11 (61.1)<br>7 (38.9)	**0.002**
Seizure frequency		Median 0<br>(IQR 0, 2)	Median 1.5<br>(IQR 0, 2)	**0.04**
Common mental disorder symptoms	Total SRQ-20 score	Median 6<br>(IQR 3, 11)	Median 10<br>(IQR 6, 14)	0.13
Risk of alcohol use (ASSIST score)	Low (ASSIST < 10)<br>Moderate to high (ASSIST > 11)	120 (67.8%)<br>57 (32.2%)	6(60%)<br>4(40%)	0.73
Quality of Life	Total QOLIE-10 score	Median 43.5 (IQR 29.7, 67.1)	Median 32.4<br>(IQR 9, 39.1)	0.**01**
Disability	Total WHODAS score	Median 11.11 (IQR 5.6, 27.8)	Median 27.78	**0.001**

			(IQR 16.7, 41.7)	

IQR = interquartile range (25th and 75th centiles indicated in the brackets); SRQ = ; QOLIE = ; WHODAS =
