# Supplementary material for: Impact of co-morbid common mental disorder symptoms in people with epilepsy in Ethiopia on quality of life and functional disability: a cohort study
Source: Glob Ment Health (Camb). 2025 Feb 26;12:e33. doi: 10.1017/gmh.2025.24 (PMC11949734; doi:10.1017/gmh.2025.24)
Supplement: Tsigebrhan et al. supplementary material 3 — Tsigebrhan et al. supplementary material [file S205442512500024Xsup003.pdf]

Supplementary file 3- measurement model

Confirmatory factor analysis for the latent variables

1. Common mental disorder (CMD) symptoms

The fit indices for CMD symptoms has indicated adequate fit of the data by $\chi 2$ = 480.1, ($p<0.0001$), CFI = 0.97, TLI = 0.97, SRMR=0.12 and RMSEA = 0.08

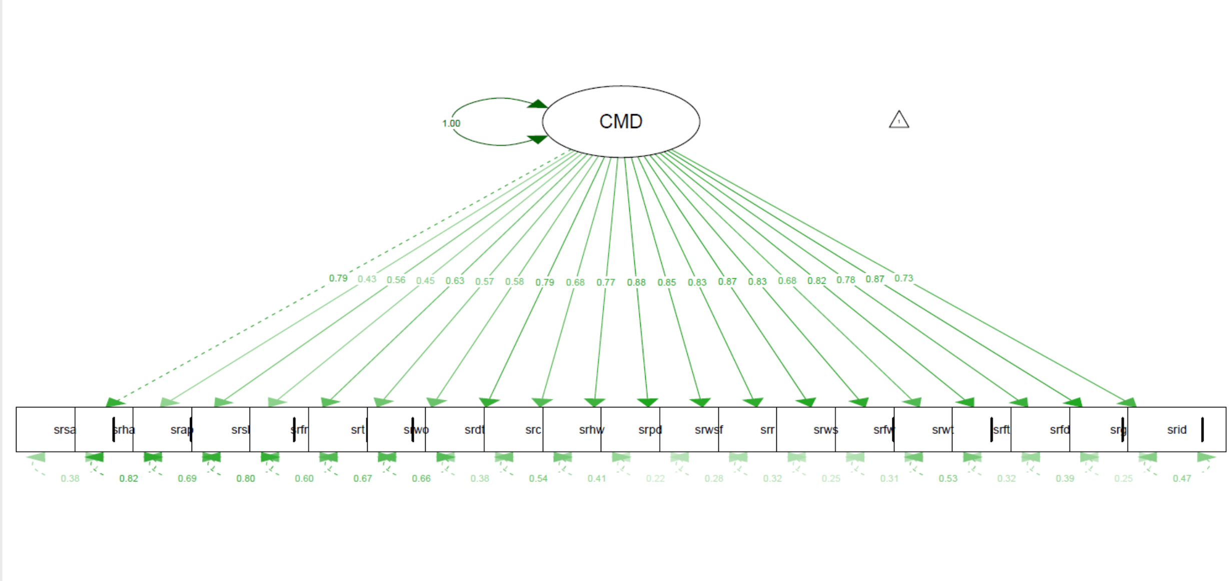

Item		Factor loading	95% Confidence interval
1	Do you often have headaches?	0.55	0.37, 0.72
2	Is your appetite poor?	0.71	0.54, 0.88
3	Do you sleep badly?	0.57	0.38, 0.76
4	Are you easily frightened?	0.80	0.64, 0.96
5	Do your hands shake?	0.73	0.56, 0.89
6	Do you feel nervous, tense or worried?	0.74	0.58, 0.90
7	Is your digestion poor?	0.92	0.77, 1.08
8	Do you have trouble thinking clearly?	1.00	0.87, 1.14

9	Do you feel unhappy?	1	1
10	Do you cry more than the usual?	0.86	0.69, 1.14
11	Do you find it difficult to enjoy your daily activities?	0.97	0.83, 1.11
12	Do you find it difficult to make decisions?	1.13	1.0, 1.25
13	Is your daily work suffering?	1.08	0.94, 1.21
14	Are you unable to play useful part in your life?	1.06	0.93, 1.18
15	Have you lost interest in things?	1.10	0.98, 1.22
16	Do you feel that you are a worthless person?	1.06	0.93, 1.19
17	Has the thought of ending your life been on your mind?	0.87	0.70, 1.04
18	Do you feel tired all the time?	1.05	0.91, 1.18
19	Do you uncomfortable feeling in your stomach?	0.99	0.85, 1.15
20	Do you get easily tired?	1.10	0.98, 1.23

2. Epilepsy related stigma

The fit indices for CMD symptoms has indicated adequate fit of the data by $\chi 2$ = 240.5, ($p<0.0001$), CFI = 0.98, TLI = 0.99, SRMR=0.08 and RMSEA = 0.07.

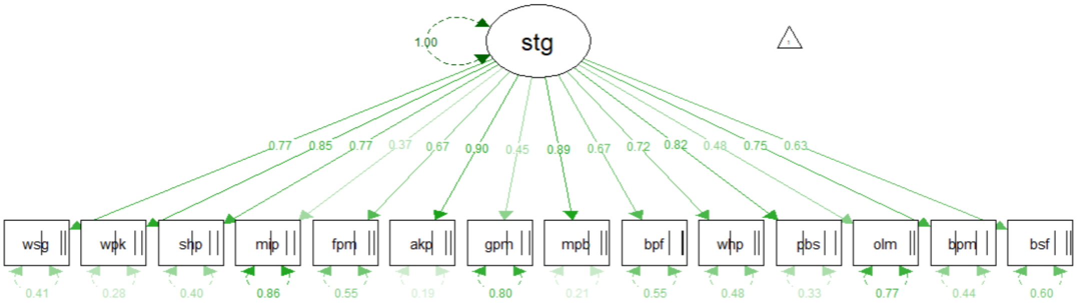

**Item**		**Factor loading**	**95% Confidence interval**
**1**	You worried that your neighbors would treat you differently	0.77	0.70, 0.84
**2**	You spent time worrying whether people would [illegible] out about it	0.85	0.80, 0.90
**3**	You sometimes felt the need to hide this fact	0.77	0.71, 0.84
**4**	You have helped other people to understand what it is like to have a psychiatric problems	0.39	0.25, 0.49
**5**	When you met people for the first time, you made a special effort to keep this fact a secret	0.67	0.58, 0.76
**6**	You worried that friends and neighbors would avoid you after they found out about it	0.90	0.85, 0.95
**7**	You have found yourself explaining to others that you are not like their picture of “crazy” people	0.45	0.34, 0.57
**8**	You worried that people would blame you for your problems	0.89	0.84, 0.94
**9**	You worried that a person looking to marry would be reluctant to marry to you	0.67	0.58, 0.77

**10**	You worried about getting out	0.72	0.64, 0.80
**11**	You felt ashamed or embarrassed about it	0.82	0.76, 0.88
**12**	You sought out people who also have a psychiatric problems	0.48	0.32, 0.64
**13**	You felt grief or depression because of it	0.75	0.68, 0.82
**14**	You felt somehow it might be your fault	0.64	0.54, 0.73

3. **Quality of life for Epilepsy**

The fit indices for quality of life latent construct has indicated adequate fit of the data by $\chi 2$ = 103.5, ($p<0.0001$), CFI = 0.99, TLI = 0.99, SRMR=0.06 and RMSEA = 0.06

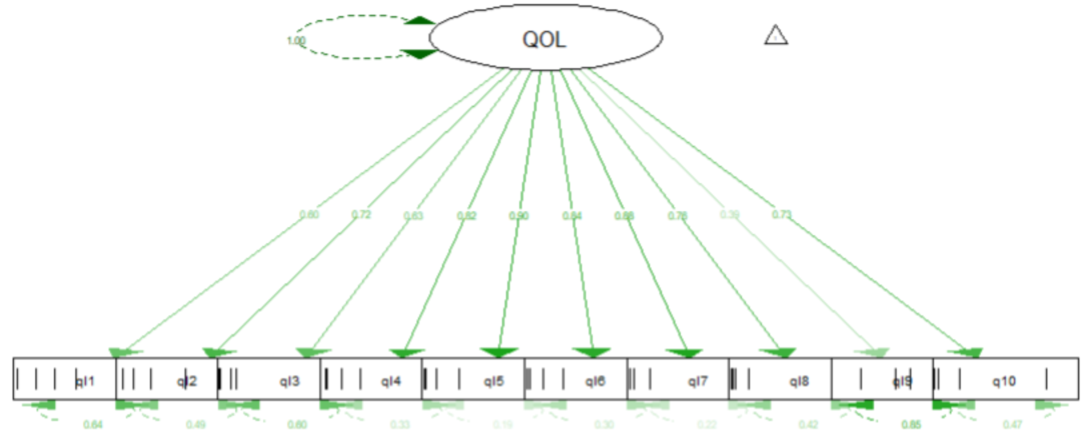

Item		Factor loadings	95% confidence interval
1	Did you have a lot of energy?	0.60	0.52, 0.68
2	Have you felt downhearted and low?	0.72	0.63, 0.81
3	How much of the time during the past 4 weeks your epilepsy or antiepileptic drugs have caused trouble with driving (or other transportation)	0.63	0.49, 0.78
	**During the past 4 weeks…**		
4	How much do your **work limitations** bother you?	0.82	0.75, 0.89
5	How much do your **social limitation** bother you?	0.90	0.85, 0.95
6	How much do your **memory difficulties** bother you?	0.84	0.77, 0.91
7	How much do **physical effects** of antiepileptic drugs bother you?	0.88	0.83, 0.94
8	How much do **psychological effects** of antiepileptic drugs bother you?	0.77	0.68, 0.85
9	How **afraid** are you of having a seizure during the next 4 weeks?	0.39	0.27, 0.51
10	How has your QUALITY OF LIFE been during the past 4 weeks (that is, how have things been going for you)?	0.73	0.66, 0.79

4. Functional disability

The fit indices for quality of life latent construct has indicated adequate fit of the data by $\chi2$ = 90.71, (p=*0.001*), CFI = 1.0, TLI = 1.0, SRMR=0.07 and RMSEA = 0.03

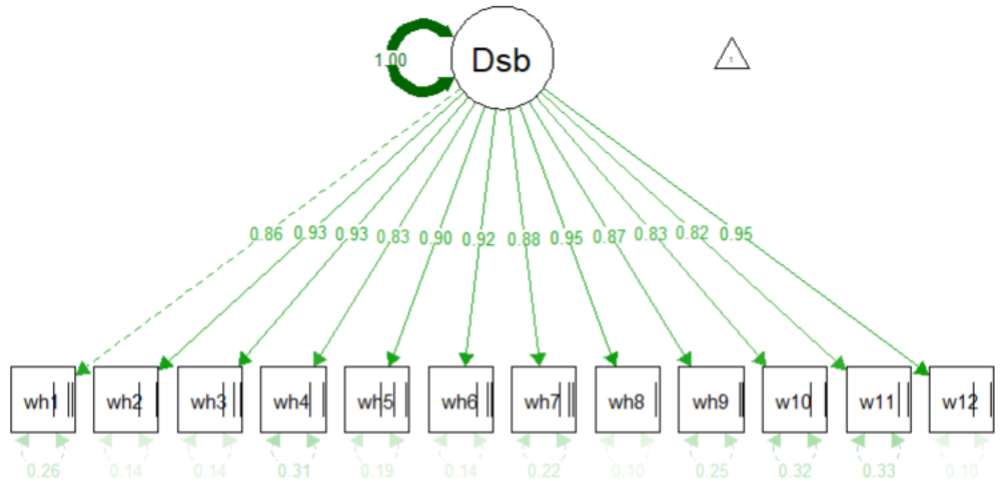

Item		Factor loading	95% Confidence interval
1	How do you rate your overall health in the past 30 days?	1	1
2	Standing for long periods such as 30 minutes?	1.08	1.0, 1.16
3	Taking care of your household responsibilities?	1.08	1.00, 1.16
4	Learning a new task, for example, learning how to get to a new place?	0.97	0.88, 1.06
5	How much of a problem did you have in joining community activities (for example, festivities, religious or other activities) in the same way as anyone else can?	1.05	0.97, 1.13
6	How much have you been emotionally affected by your health problems?	1.08	1.0, 1.16
	In the last 30 days, how much difficulty did you have in:		
7	Concentrating on doing something for 10	1.03	0.95, 1.11

	minutes?		
8	Walking a long distance such as a kilometer?	1.10	1.02, 1.19
9	Washing your whole body?	1.01	0.92, 1.11
10	Getting dressed	0.96	0.85, 1.05
11	Dealing with people you do not know?	0.95	0.85, 1.05
12	Maintaining a friendship?	1.10	1.03, 1.18
