# Supplementary material for: Impact of co-morbid common mental disorder symptoms in people with epilepsy in Ethiopia on quality of life and functional disability: a cohort study
Source: Glob Ment Health (Camb). 2025 Feb 26;12:e33. doi: 10.1017/gmh.2025.24 (PMC11949734; doi:10.1017/gmh.2025.24)
Supplement: Tsigebrhan et al. supplementary material 4 — Tsigebrhan et al. supplementary material [file S205442512500024Xsup004.pdf]

Supplementary file 4

SEM after imputation of the variables with missed values (quality of life, social support and seizure frequency in the last 6 months)

The fit indices for the full structural model has indicated adequate fit of the data by $\chi 2$ = 162.8, ($p<0.0001$), CFI = 0.97, TLI = 0.97, SRMR=0.10 and RMSEA = 0.07.

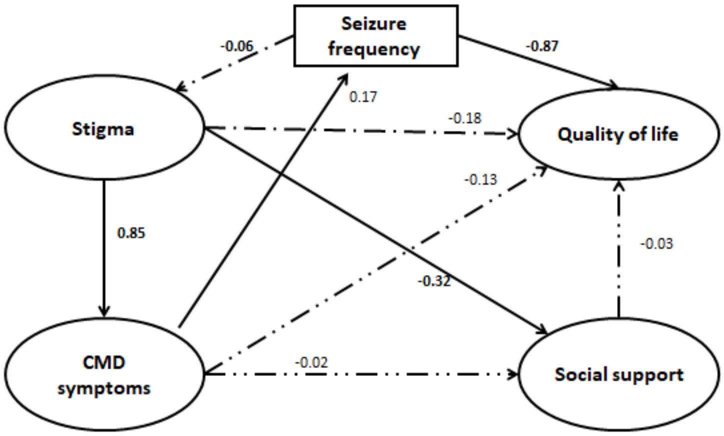

Figure 1. SEM after imputation of the missing values for quality of life (QOL) score and social support (SOS) and seizure frequency in the 6 month follow up
